# Pregnancy denial and early infant development: a case-control observational prospective study

**DOI:** 10.1186/s40359-019-0290-3

**Published:** 2019-04-11

**Authors:** Julie Auer, Coralie Barbe, Anne-Laure Sutter, Dominique Dallay, Laurianne Vulliez, Didier Riethmuller, Violaine Gubler, Valérie Verlomme, Stéphanie Saad-Saint-Gilles, Alain Miton, Emmanuelle Tessier, Olivier Parant, Julie Le Foll, Agnès Bourgeois-Moine, Sylvie Viaux, Marc Dommergues, Gisèle Apter, Joëlle Belaisch-Allart, Anne Danion, Israël Nisand, Olivier Graesslin, Alexandre Novo, Julien Eutrope, Anne-Catherine Rolland

**Affiliations:** 1 Service de Psychothérapie de l’enfant et de l’adolescent, Pôle Femme-Parents-Enfant, Av du Gl Koenig, Centre Hospitalier Universitaire, 51092 Reims Cedex, France; 2 Unité d’aide méthodologique, Hôpital Robert Debré, Av du Gl Koenig – CHU, Reims, France; 3 Réseau de psychiatrie périnatale, Pôle Universitaire de Psychiatrie Adulte - Hôpital Charles Perrens, 121, rue de la Béchade, 33076 Bordeaux, France; 4 Maternité Pellegrin, Place Amélie Raba-Léon, 33076 Bordeaux cedex, France; 5 Psychiatrie infanto-juvénile - Centre Hospitalier Régional Universitaire, Hôpital Saint-Jacques, 2 place Saint-Jacques, 25030 Besançon cedex, France; 6 Service de Gynécologie Obstétrique, CHU Besançon -Hôpital Jean Minjoz, 25000 Besançon, France; 7 Aubagne, France; 8 C.H.I.T.S. Hôpital Sainte Musse, 54 Henri Sainte Claire Deville, 83056 Toulon, France; 9 CPN Laxou – Nancy, 1 rue Voltaire, 54300 Luneville, France; 10 Maternité Régionale, 10 Avenue Dr Heydenreich, 54000 Nancy, France; 11 Service de Psychiatrie de l’Enfant et de l’Adolescent - Pôle Psychiatrie, Hôpital La Grave, Place Lange, 31059 Toulouse Cedex 9, France; 12 Pôle de Gynécologie Obstétrique et Médecine de la Reproduction, Hôpital Paule de Viguier - CHU de Toulouse, 330 avenue de Grande-Bretagne, 31059 Toulouse Cedex 9, France; 13 Polyclinique Ney, Hôpital Bichat, 124 Bd Ney, Paris 18ième, France; 14 Service de gynécologie obstétrique, Hôpital Bichat, 46 Rue Henri Huchard, 75877 Paris, France; 15 UPEP Vivaldi, Hôpitaux Universitaires Pitié Salpêtrière, GHU Pitié-Salpêtrière, 47, boulevard de l’Hôpital, 75013 Paris, France; 16 Service de Gynécologie Obstétrique, Groupe Hospitalier Pitié-Salpêtrière, 47-83 Bd de l’Hôpital, 75651 Paris Cedex 13, France; 17 Unité de Psychiatrie Périnatale d’Urgence Mobile en Maternité Service, EPS Erasme, 14, rue de l’Abbaye, BP 10081, 92161 Antony cedex, France; 18 Service de Gynécologie-Obstétrique et Médecine de la Reproduction, Centre Hospitalier des Quatre Villes, 141, Grande Rue, 92318 Sèvres, France; 19 Service de Psychiatrie de l’Enfant et de l’Adolescent, Pôle de Psychiatrie, Santé Mentale et Addictologie, Hôpitaux Universitaires de Strasbourg, 1 Place de l’Hôpital, 67091 Strasbourg, France; 20 Pôle de gynécologie-obstétrique, Hôpital de Hautepierre, Avenue Molière, 67200 Strasbourg, France; 21 Service de gynécologie-obstétrique – Pôle Femme-Parents-Enfant, Hôpital Maison Blanche, 45 Rue Cognacq Jay, 51092 Reims, France

## Abstract

**Background:**

The denial of pregnancy is the non-recognition of the state of the current pregnancy by a pregnant woman. It lasts for a few months or for the whole pregnancy, with generally few physical transformations. In this study, we will consider the denial of pregnancy as a late declaration of pregnancy (beyond 20 weeks of gestation) as well as a lack of objective perceptions of this pregnancy. The main objective of this study is to explore the relationship between pregnancy denial and the development of the infant (attachment pattern of the infant, early interactions of mother-infant dyads, and early development of the infant).

**Methods:**

The design is a case-control prospective study, which will compare two groups of mother-infant dyads: a “case” group with maternal denials of pregnancy and a “control” group without denials of pregnancy. A total of 140 dyads (mother + infant) will be included in this study (70 cases and 70 controls) and followed for 18 months. The setting is a national recruitment setting with 10 centers distributed all over France. The follow-up of the “cases” and the “controls” will be identical and will occur over 5 visits. It will include measures of the infant attachment pattern, the quality of early mother-infant interaction and infant development.

**Discussion:**

This study aims to examine the pathogenesis of pregnancy denial as well as its consequences on early infant development and early mother-infant interaction.

**Trial registration:**

Clinical Trial Number: NCT02867579 on the date of 16 August 2016 (retrospectively registered).

## Background

### Definition of pregnancy denial

Described in the 1970s [1, 2], pregnancy denial occurs as the unconsciousness of being pregnant for several months or throughout the entire period of pregnancy. Usually, body transformations are not clearly noticeable. The prevalence of this symptom is estimated to be 1 case of denied pregnancy in 475 births [3].

There is no consensus concerning the definition of pregnancy denial. First, there is no consensus on the threshold date from when the pregnancy is considered denied if unacknowledged. On the one hand, some authors consider that the threshold date is beyond the first trimester: 14 weeks of amenorrhea [4, 5], beyond 21 weeks of amenorrhea [6] or beyond 20 weeks of pregnancy [3]. On the other hand, some authors consider a much longer duration. For example, Friedman proposed the end of the third trimester as threshold date [7]. Second, besides duration, denial may be incomplete. Two types of pregnancy denial have been proposed: partial denial with late pregnancy discovery (after 5 month of pregnancy) and total denial with pregnancy discovery while delivering [8]. Third, the encountered terminologies differ from author to another, including pregnancy denial and pregnancy negation [9]. Dayan describes pregnancy negation as “*a large range of occurrences, which are the refusal or incapacity of a pregnant woman to admit her condition”* [10]. These difficulties in properly defining pregnancy denial reflect the clinical heterogeneity of the patients.

The denial of pregnancy calls into question maternal psychological functioning. However, to date, no link between any specific psychiatric disorder and denial of pregnancy has been established [11]. During our clinical meetings, mothers who presented a denial of pregnancy report a difficult personal history with many breaks and events described as traumatic.

### Consequence of pregnancy denial

During pregnancy, a mother is getting prepared to meet her child and build quality interactions through a maturational process leading to a psychological reorganization. The pregnancy and the birth represent, for the woman, an essential phase of her psycho-affective development, comparable to the adolescence in its somatic, hormonal and psychological changes. In the past, many authors have studied these psychological reorganizations and proposed theories [12, 13]. These psychological reorganizations enable the mother to adapt to her new role and to create a containing and reassuring environment for her child. For women prone to pregnancy denial, this period of psychological reorganization is almost non-existent. The first part of the pregnancy’s story is lacking. More recently, a study found that perceiving frequent fetal movements was associated with higher scores of prenatal attachment [14]. Several publications [15–20] report observations of cases of pregnancy denial. Only one retrospective studies focused on the future of the child [21]. To our knowledge, no prospective study has focused on the future of the mother-infant relationship.

## Objectives

### Study hypothesis

We put forward the following hypothesis: an insecure attachment of the mother would participate or at least would increase the likeliness of pregnancy denial. These mothers would find it difficult (even impossible in some cases) to access the experiences of their infants, which are essential in the psychological reorganizations necessary during pregnancy to prepare the woman to accept her new functions as a mother.

Moreover, the absence or reduction of the duration of the usual 9-month period for psychological elaboration during the pregnancy calls into question its potential impact on the quality of mother-child interactions. Are there consequences on the infant’s development as well as on his pattern of attachment? We hypothesize that the attachment and the development of the infant to be born, as well as the quality of mother-infant interaction, are disturbed when the woman presents a denial of pregnancy. Specifically, we expect these consequences to be stronger with a longer duration of pregnancy denial.

### Study objectives

The primary objective of this study is to examine the relationship between pregnancy denial and infant attachment patterns, early mother-infant interaction and early infant development.

The secondary objectives of this research are as follows:

- To explore among women with pregnancy denial the influence of the duration period between the pregnancy announcement and delivery on infant attachment pattern, early mother-infant interaction and early infant development;
- To study the factors associated with pregnancy denial, including the type of maternal attachment and the existence of a maternal personality disorder and/or a psychiatric condition.

## Methods

### Trials status

This study has been retrospectively registered in the European registry (EudraCT 2011-A01498-33) and in clinicaltrials.gov (NCT02867579). The recruitments and the interventions started on April 2013 and will be completed in April 2019.

### Study design and setting

This study entitled “Attachment and pregnancy denial” is a national multicenter prospective case-control study with 13 French investigation centers (Anthony Ile-de-France, Besançon, Bordeaux, Nancy, Paris Bichat, Paris La Pitié Salpêtrière, Reims, Strasbourg, Toulouse, Amiens, Troyes, Aubagne, Lille).

### Population

*Inclusion criteria* are as follows. In the case group, dyads are composed of a woman with pregnancy denial and her infant. Pregnancy denial will be defined by a pregnancy announcement after 20 weeks of gestation and a lack of objective perceptions of the pregnancy by the woman. Women with no follow-up of the pregnancy due to geographical, social or administrative reasons (absence of insurance coverage, family conflicts, unemployment, and pregnancy hidden to the employer) will not be included. In the control group, dyads are composed of a woman without pregnancy denial and her infant. The case group and the control group will be matched on primiparous and non-primiparous mothers and on premature (birth before 37 GW) and non-premature infants.

*Exclusion criteria* are as follows: minor women (< 18 years old), women with intellectual disability, women with an acute or chronic psychotic condition, women who do not speak fluent French, women with illegal administrative status, newborn with a life-threatening prognosis, newborn with an organic malformation and/or genetic abnormality observed before leaving the hospital, and medically assisted reproduction (for the control group).

In a pragmatic way, due to the lack of data concerning the relationship between pregnancy denial and infant attachment pattern in the literature, participation will be proposed to all women with pregnancy denial during the inclusion period. Given the average number of births per year and by center (2500 on average), the number of participating centers (N=13), the frequency of pregnancy denial (2/1000 child births), the duration of the inclusion phase (48 months) and the expected participation acceptance rate (25%), 70 women with pregnancy denial will be included in this study. Therefore, 140 dyads mother-infant women will be included in this study. Based on the hypothesis of a score for disorganization [22, 23] of 3.44 ± 1.90 in the control group, inclusion of 70 dyads mother-infant per group (and evaluation of 50 dyads at 18 months because of lost of follow up) will highlight a score for disorganization of 2.37 in the case group, with a signification of 5%, a power of 90% and a bilateral (NQuery Software 7.0).

### Prospective visit calendar

Participation to the study will be proposed during hospitalization after delivery. The follow-up of the cases and the control will be similar. The total duration of participation of each dyad is 20 months. The design of the study will not change the eventual care provided to the dyad. If some care is proposed or given, a description of this care will be included in the data collection.

The study’s follow-up includes 6 visits (Table 1).

**Table 1:** Visits description and data collected

Timepoint	1st visit Inclusion	2nd visit	3rd visit	4th visit	5th visit	6th visit Thanks & Survey
	Maternity	Obstetrical Consultation	Home	Home	Psychological Laboratory	Home
	S 1	S 6–8	M 6	M 12	M 18	M20
ENROLMENT	X					
- Inclusion criteria	X					
- Non-Inclusion criteria	X					
- Informed consent	X					
ASSESSMENTS	X					
- Demographic information	X					
- Social information	X					
- Medical information	x					
- MINI^a^ (mother)	X					
- BDI^b^ (mother)	X			X		
- STAI^c^ (mother)	X			X		
- PSSQ^d^ (mother)	X			X		
- EPDS^e^ (mother)		X				
- AAN^f^ (mother)		X				
- DSST^g^ (infant)			X	X	X	
- CIB^h^ (dyad)			X	X		
- QT6^i^ (mother)			X			
- ADBB^j^ scale (infant)			X	X		
- IPDE^k^ (mother)			X			
- SSP^l^ (infant)					X	

^a^MINI: Mini International Neuropsychiatric Interview

^b^BDI: Beck Depression Inventory

^c^STAI: Scale Trait Anxiety Inventory

^d^PSSQ: Perceived Social Support Questionnaire

^e^EPDS: Edinburgh Postnatal Depression Scale

^f^AAN: Adult Attachment Narratives

^g^DSST: Denver Developmental Screening Test

^h^CIB: Coding Interactive Behaviour

^i^QT6: Questionnaire on the 6 months old infant’s Temperament

^j^ADDB scale: Alarm Distress BaBy scale

^k^IPDE: International Personality Disorders Examination

^l^SSP: Strange Situation Procedure

During the first visit (during the week after delivery), informed consent, data (demographics, age, marital status, level of education, financial resources, socio-economic data of the child’s father, developmental data of the child’s siblings) will be collected. Additionally, medical information, including gestation and parity, pregnancy records, and medical, surgical and psychiatric history, will be extracted from the mother’s charts. Direct assessment of perceived social support with the Perceived Social Support Questionnaire (PSSQ) [24] and psychiatric condition will be provided with the Mini International Neuropsychiatric Interview (MINI) for Psychiatric Disorders Axis I [25].

The second visit (6 to 8 weeks after delivery) includes the following: assessments of depression with the Beck Depression Inventory (BDI) [26] the Edinburgh Perinatal Depression Scale EPDS [27], of maternal anxiety with the Scale Trait Anxiety Inventory (STAI) [28] of personality disorders using the screening of the International Personality Disorders Examination (IPDE) [29] and of maternal attachment patterns with the Adult Attachment Narratives (AAN) [30].

The third visit (6 months after delivery) includes assessments of mother-infant interaction during a meal with the Coding Interaction Behavior system (CIB) [31, 32], of the child’s temperament with a questionnaire on the 6-month-old infant’s temperament (QT6) [33, 34], of maternal depression (EPDS) [27] of child development using the Denver Developmental Screening Test [35] and of the relational retreat behavior of the child with the Alarm Distress Baby (ADBB) Scale [36] and the semi-structured interview for personality disorders (IPDE) [29].

The fourth visit (12 months after delivery) includes evaluations of maternal depression (BDI and EPDS), of maternal anxiety (STAI), of perceived social support (PSSQ), of interactions during a meal (CIB), of child development (DSST) and of the relational retreat behavior of the child (ADBB).

The fifth visit (18 months after delivery) includes evaluations of the infant’s pattern of attachment with the SSP (Strange Situation Procedure) [35] and of the infant’s development (DSST).

The sixth visit (20 months after birth) is a free assessment with the objective of thanking the parents for their participation and surveying them about the organization and design of this study.

### Measures

For the infants: The attachment pattern of the infant is assessed with the Strange Situation Procedure [37, 38] A mother and an 18-month-old infant are under observation in a laboratory. The observer notes the child’s reactions during 8 episodes of 3 minutes involving the separations and reunions between the mother and the child, as well as the introduction of a stranger. The situation is videotaped. The coding system of interactive behaviors allows a categorization into 4 attachment patterns: “secure”, “anxious-avoidant insecure”, “anxious-resistant insecure” and “disorganized/disoriented insecure” (regrouped into two categories: “secure” and “insecure”). Richters and associates [39] developed a method to score attachment in a continuous way. Van IJzendoorn and Kroonenberg [23] adapted and validated the algorithm for use with Strange Situation interactive scales without scores for crying. The resulting algorithm yields a continuous score for attachment that is strongly associated with the insecure vs. secure attachment classifications. Higher security scores indicate a more secure attachment relationship. Continuous scores for disorganization were derived directly from coding the conventional 9-point scale for disorganization [40], with higher scores indicating more disorganized behavior.

The quality of early mother-infant interaction is assessed with the Coding Interactive Behavior (CIB) of Feldman [31, 32]. The CIB includes 42 items (21 concerning the mother, 16 concerning the child and 5 concerning the dyad). Each item is evaluated on a 5-point scale. During the first year, 6 dimensions are extracted from the CIB items: (i) parental sensitivity, (ii) parental intrusion, (iii) infant’s social engagement, (iv) infant’s negative emotionality/infant’s engagement, (v) dyadic reciprocity and (vi) dyad’s negative states. The scale consists of 2 segmentations of 15 minutes of interactions: one session of a game and one session of feeding. The 6-month-old infant’s temperament is assessed with the French version of the Infant Characteristics Questionnaire of Bates [33]. The QT6 is a self-questionnaire administered to the mothers with 26 questions about the “difficult” temperament of the infant. It was validated in France with 794 mothers of infants aged 6 to 9 months [34].

The infant’s development is evaluated with the Denver Developmental Screening Test [35]. The child level is assessed in different areas, such as gross motor skills, language, fine motor skills, and social contact.

Social contact is also evaluated on the Alarm Distress Baby Scale [36]. This scale includes 8 items concerning facial expression, eye contact, body activity, self-simulation gestures and finger activities, level of vocal expression, liveliness of a response to stimulation, ability to connect with someone else and attractiveness.

For the mothers: The representations of the adult’s attachment are studied with the Adult Attachment Narrative [30] through the analysis of 4 narratives (2 referring to relations among adults, and 2 in mother-child relations) built by the subject. The adult is asked to create a story using 12 words presented by the examiner (these words establishing the weft of the history). These narratives are blind-recorded and analyzed by trained professionals, and every narrative receives a note from 1 to 7 reflecting the secure base of the script in connection with the pattern of attachment of the subject. The average obtained on these 4 narratives estimates the security of the attachment of the subject and allows for the categorization into 2 groups: notes ≥ 3, insecure pattern of attachment; and notes > 3, secure patterns of attachment. The interview lasts approximately half an hour. The entire interview is transposed verbatim to allow for quotations.

The evaluation of personality disorders described in the ICD-10 is completed with the International Personality Disorders Examination (IPDE) [29]. This semi-structured diagnostic interview contains 67 items. The criteria of personality are grouped into six domains: work, personal, interpersonal relations, affects, apprehension of reality and control of impulses. The length of administration of the instrument varies between 60 and 90 minutes.

The main psychiatric disorders of the axis I of the ICD-10 are explored with the Mini International Neuropsychiatric Interview (MINI) [25]. This structured diagnostic maintenance tool contains 120 questions and is divided into 16 modules, each corresponding to a diagnosis category. The MINI was simultaneously developed in French and in English.

The mothers’ depressive feelings are evaluated with two scales. First, the Edinburgh Postnatal Depression Scale [27] a quick postnatal-specific self-questionnaire screening for postnatal depression, has been validated by a number of studies. Second, the Beck Depression Inventory [26] includes 21 items of symptoms and attitudes that describe a specific depressive behavior, with response options ranging from 0 to 3 by a series of 4 statements reflecting the degree of severity of the symptom.

The mothers’ anxious feelings are evaluated with Spielberger’s State-Trait Anxiety Inventory Form Y [28]. The inventory is intended to estimate, on the one hand, trait anxiety, and on the other hand, state anxiety, through 20 items that only focus on the psychological and not the somatic aspects of anxiety. The Y version was developed to eliminate items more bound to depression. The STAI is intended for self-administration, and every answer to an item of the questionnaire corresponds to a score from 1 to 4.

The availability of and satisfaction with the social support received are evaluated with the Perceived Social Support Questionnaire [24] a self-assessment scale that estimates the 4 main forms of social support, represented in 4 questions on the scale: the support of esteem, material or financial support, informative support, and emotional support. For each type of support, it assesses how many people dispense it, who these people are (family, friends, colleagues, professionals) and if the subject is satisfied with this support. Two scores are obtained for every subject: availability (number of people having participated in the support) and received satisfaction ("quality") of this support.

### Bias

We expect a percentage of women to drop out of the study before its end, especially in the case group. Mothers who experience a denial of pregnancy are more likely to drop out of the study earlier than mothers in the control group. This early attrition will impact the size of our sample.

### Coding

The attachment pattern of the mother (AAN) and of the infant (Strange Situation) and the mother-infant interactions (CIB) will be all evaluated based on videos conducted by each center. Ratings will be performed by one single qualified team (principal investigator center) and by two trained raters who will remain blind to group status.

### Statistical methods

Data will be described using means and standard deviations for quantitative variables and numbers and percentages for qualitative variable. Normal distributions will be checked.

To study factors associated with pregnancy denial, characteristics of women with pregnancy denial and characteristics of women without pregnancy denial will be compared with univariate analysis (using Student’s t, Wilcoxon, chi-square or Fisher’s exact tests, as appropriate) and multivariate analysis (logistic regression).

Factors associated with the attachment pattern of the child, divided into two categories (“secure” and “insecure”), will be studied by univariate (Student’s t, Wilcoxon, chi-square or Fisher’s exact tests, as appropriate) and multivariate analysis (logistic regression).

A *P*-value <0.05 will be considered statistically significant.

All analysis will be performed using SAS version 9.4 (SAS Inc., Cary, NC, USA).

## Discussion

This study aims to examine the pathogenesis of pregnancy denial as well as its consequences on the attachment pattern of the child, the quality of early mother-infant interaction and infant development. We expect this study to more clearly define what pregnancy denial is and, thus, to improve the care of mother-infant dyads.

### Strengths and limitations of this study

- The denial of pregnancy remains a poorly understood symptom, and there are few publications and no prospective studies on this topic, especially case-control studies.
- This is the first study exploring the relationship between pregnancy denial and the development of the infant.
- This is the first study, influenced by attachment theory, to evaluate and compare the attachment patterns of the mothers and infants in case and control groups.
- This study did not limit mothers’ access to specific care, if indicated.
- Mothers who experienced a denial of pregnancy are expected to drop out of the study earlier than mothers in the control group, which will impact the size of our sample as well as the results of the study.

## Abbreviations

AAN: Adult Attachment Narratives
ADBB: Alarm Distress BaBy
BDI: Beck Depression Inventory
CIB: Coding Interaction Behavior system
DSST: Denver Developmental Screening Test
EPDS: Edinburgh Perinatal Depression Scale
GW: Gestational Weeks
ICD-10: International Classification of Diseases 10th
IPDE: International Personality Disorders Examination
MINI: Mini International Neuropsychiatric Interview
PSSQ: Perceived Social Support Questionnaire
QT6: Questionnaire on the 6-month-old infant’s Temperament
SSP: Strange Situation Procedure
STAI: Scale Trait Anxiety Inventory
